# Cognitive bias analysis of young novice drivers’ observation abilities—A questionnaire-based study

**DOI:** 10.1371/journal.pone.0251195

**Published:** 2021-05-11

**Authors:** Wang Xiang, Xuemei Liu, Qunjie Peng, Qingwan Xue, Wei Hao, Ji Yu

**Affiliations:** 1 Hunan Key Laboratory of Smart Roadway and Cooperative Vehicle-Infrastructure Systems, Changsha University of Science & Technology, Changsha, Hunan, China; 2 Shenzhen Transportation Design & Research Institute CO., LTD, Shenzhen, Guangdong, China; 3 Beijing Key Laboratory of Urban Intelligent Traffic Control Technology, North China University of Technology, Beijing, China; Tongii University, CHINA

## Abstract

Observation ability, which is the basis of following decision-making and vehicle manipulation behaviour, is of great importance while driving. However, the subject self-cognition and objective assessment of driving ability are usually different, especially for the young novice drivers. In this paper, drivers’ observation abilities for both static traffic signs and markings and dynamic surrounding vehicles were investigated based on questionnaire data. Effects of gender and driving characteristics (driving year, driving frequency, driving time) on drivers’ observation abilities were verified by ANOVA analysis and structural equation model (SEM) from two aspects: drivers’ self-assessment scores (self-assessment) and mutual assessment scores (evaluated by others). Significant difference could be found between all the factors and drivers’ self-assessment scores, while only driving year had a significant effect on drivers’ mutual assessment scores. Besides, cognitive bias was found between all the driving year groups. It seemed that drivers with driving experience less than one year were always overconfident with their driving abilities. And drivers with driving experience more than three years usually gave the most conservative assessment scores for themselves and others. With more exposures to various traffic conditions, experienced drivers are more aware of their limitations on observing surrounding information, while young novice drivers still not realized their limitations on observing traffic signs and other vehicles in a right way.

## 1. Introduction

During the past ten years, there has been a surge in number of young drivers in China. It was estimated that the number of young novice drivers (with a driving license less than 3 years) was 89 million, accounting for 24% among all the drivers in 2018. Young novice drivers have a higher crash risk than all the other driving age due to their lack of driving experience [1, 2]. Thus, novice drivers have become an important factor affecting road safety and efficiency due to the great amount and lacking of driving experience [3].

In fact, driving is a complex dynamic process-control activity that consists of observation, cognition, thought and decision, and operation functions. Among which, observation is the first step and all the following activities are conducted based on observation [4, 5]. For novice drivers, because of insecurity and mental stress, they normally look straight ahead, while some important information around, e.g. traffic signs, and may be ignored. Therefore, their effective vision fields are narrower than experienced drivers [6]. In addition, due to their fewer exposures to complex road scenarios, young novice drivers’ normally have a lower awareness of the potential risks, compared to experienced drivers [7, 8]. Studies have found that novice drivers’ observation abilities could be alleviated significantly by improving their driving experience [9]. Following driving practice, more and more useful information including both static road environment and dynamic traffic participants can be noticed by novice drivers [10]. Although novice drivers’ observation ability can be enhanced by practice, their self-cognition of driving abilities cannot be improved by this way [11].

Self-cognition of driving ability is an important factor which may affect drivers’ driving decision and their vehicle control behaviour [12]. But drivers are usually not able to make exact judgments during driving, i.e., judgment errors or inconsistencies between the judgment itself and the real traffic situation may occur, which is called cognitive bias. Cognitive bias of driving ability has been widely approved in previous studies [13, 14]. It was shown that many factors can affect drivers’ self-cognition of driving ability, such as age, gender, education, and driving practice [15, 16]. Results generated due to cognitive bias can be categorized into two types: one is underestimation and the other one is overestimation. For underestimation, drivers’ vehicle control behaviour tends to be conservative, e.g., keeping at a low driving speed; for overestimation, drivers usually keep a positive attitude towards the current traffic situation, which make their driving behaviour radical, e.g., speeding, frequently changing lanes [17, 18]. Although conservative drivers may have a lower crash risk compared with aggressive drivers, the traffic efficiency cannot be ensured.

For drivers’ psychology, accurate perception of own driving skill is especially desirable for traffic safety [19], since by knowing one’s own strengths and weaknesses, one can take efficient compensatory action to reduce collision risk [20]. Therefore, eliminate cognitive bias or reduce cognitive bias in some extents is of great importance. The purpose of this paper is to investigate the cognitive bias (overconfidence, or normal, or lack of confidence) of novice drivers’ observation abilities on both static objects (such as traffic facilities) and dynamic objects (such as surrounding vehicles). Factors such as gender and driving information (driving year, driving frequency and driving time) were considered when drivers rating the observation abilities for themselves and others. A structural equation model was built to investigate the casual relationship between each factor and drivers’ self-assessment and mutual-assessment.

## 2. Methodology

### 2.1 Participants

In this study, a total of 1031 questionnaires were collected by an online questionnaire survey. Through checking the integrity of the questionnaire answers and their relevance for similar questions, there are 569 valid questionnaires and 462 invalid questionnaires. As the aim of this study is to investigate young novice drivers’ observation abilities while driving, 435 respondents with the age between 18 and 25 years were chosen as the young novice group, and 134 drivers who were older than 25 were included in this study as the experienced drivers group. Table 1 shows the demographics information of all the participants included in this study.

**Table 1:** Demographics information of participants.

Variable	Classification	Number	Percentage
Gender	Male(M)	302	53.08%
	Female(F)	267	46.92%
Driving Year	Within 1 year (<1)	209	36.73%
	1 to 3 years (1~3)	226	39.72%
	Over 3 years (>3)	134	23.55%
Driving Frequency	1 to 2 times per week (1~2)	239	61.60%
	3 to 5 times per week (3~5)	52	13.40%
	Over 5 times per week (>5)	97	25.00%
Driving Times	Less than 30 minutes (<30)	107	27.58%
	30 to 60 minutes (30~60)	178	45.88%
	More than 60 minutes (>60)	103	26.55%

### 2.2 Methodology

The study was approved by the Ethics Committee of Changsha University of Science & Technology to conduct anonymous questionnaire survey, and no additional informed participant consent was required. The data derived from drivers’ questionnaire drafted by Dr. Wang Xiang was analyzed. Dr. Wang Xiang and Xuemei Liu in Transportation Research Center administered this questionnaire. Moreover, the details about the questionnaire can be seen in S1 Appendix.

**Fig 1:**
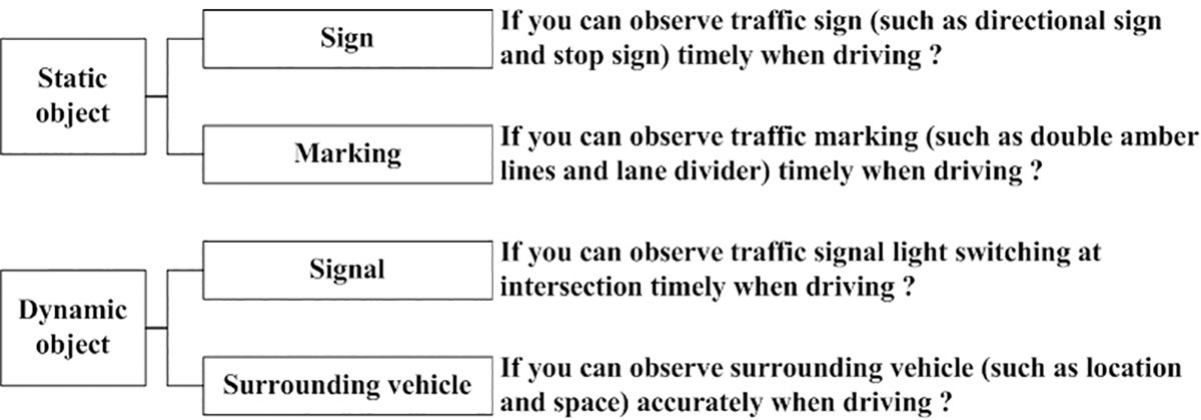
Self-assessment structure and content.

For the questionnaire adopted in this paper, participants were asked to complete two sections: one is self-assessment and the other section is mutual-assessment. For both self-assessment and mutual-assessment sections, participants had to rate their own and other’s observation abilities on a scale of one to ten from two aspects: drivers’ observation abilities of static object (road marking and signs) and dynamic object (signal and surrounding vehicles). And one referred to the weakest observation ability, and ten referred to the best observation ability. Fig 1 shows the basic structure and information included in the self-assessment survey. Similarly, each participant needed to rate other participants’ observation ability of static and dynamic information, e.g., How do you think drivers with driving experience less than one year can observe traffic signs (such as directional sign and stop sign) while driving? There are two questions for both static and dynamic information. The assessment score is the average value of two assessment scores for static (or dynamic) information. In addition, previous results were extracted as the objective driving ability based on meta-analysis to compare the self-assessment and mutual-assessment information.

The questionnaire had a high level of internal consistency as measured by Cronbach’s alpha (0.841). The significant value of Bartlett’s test of sphericity (p <0.001) and the Kaiser-Meyer-Olkin (KMO) value of 0.722 indicated that the assumptions for factor analysis were met. Based on survey data, ANOVA was adopted in this study to test and compare the difference of observation ability among demographics (gender) and driving information (e.g., driving frequency).

A structural equation model (SEM) was built to explore the direct and indirect causal effects between each factors and drivers’ observation abilities. SEM can weigh the influences of the exogenous and endogenous variables [21], which combined with factor analysis and simultaneous equation models has been widely used as a linear-in-parameter multivariate statistical modeling technique. In this study, the SEM approach was used to investigate driving cognition as revealed by the causal relationships between driving years, self-assessment and mutual-assessment. The software package IBM SPSS Analysis of Moment Structures (AMOS) 21 was used to perform SEM analyses.

Drivers’ mean self-assessment score (SAS) and mutual-assessment score (MAS) in terms of gender, driving frequency, driving year and driving time are shown in Tables 2 and 3, respectively.

**Table 2:** Descriptive statistical of mean self-assessment score.

Variable		Static objects(S) (traffic facilities)			Dynamic objects(D) (surrounding vehicles)		
		Within 1 year	1 to 3 years	Over 3 years	Within 1 year	1 to 3 years	Over 3 years
Gender	Male	7.76	8.20	8.28	7.64	8.22	8.38
	Female	6.60	6.76	7.19	6.83	6.56	6.98
Driving Year		7.21	7.59	7.9	7.26	7.52	7.89
Driving frequency (per-week)	1 to 2 times	6.87	7.36	7.47	6.98	7.20	6.74
	3 to 5 times	7.82	7.85	7.67	7.64	8.15	7.95
	Over 5 times	8.53	8.29	8.38	8.41	8.29	8.45
Driving time (minutes)	Less than 30	6.57	7.29	6.78	6.83	7.06	7.47
	30 to 60	7.27	7.56	8.02	7.29	7.54	7.57
	More than 60	7.96	8.09	8.36	7.77	8.11	8.40

**Table 3:** Descriptive statistical of mean mutual-assessment score.

Variable		Static objects (traffic facilities)			Dynamic objects (surrounding vehicles)		
		Within 1 year	1 to 3 years	Over 3 years	Within 1 year	1 to 3 years	Over 3 years
Gender	Male	6.09	7.32	8.23	5.83	7.30	8.46
	Female	5.98	7.18	8.06	5.74	7.12	8.19
Driving year	Within 1 year	6.11	7.27	8.13	5.96	7.33	8.32
	1 to 3 years	6.33	7.50	8.31	5.89	7.33	8.46
	Over 3 years	5.44	6.81	7.90	5.35	6.84	8.14
Driving frequency (per-week)	1 to 2 times	6.16	7.28	8.10	6.03	7.30	8.22
	3 to 5 times	6.23	7.48	8.19	5.73	7.13	8.58
	Over 5 times	5.89	7.14	8.08	5.58	7.07	8.43
Driving time (minutes)	Less than 30	6.14	7.21	8.02	6.13	7.36	7.22
	30 to 60	6.05	7.30	8.15	5.78	7.19	8.25
	More than 60	6.15	7.30	8.15	5.79	7.13	8.30

## 3. Results

### 3.1 Self-assessment analysis

**Fig 2:**
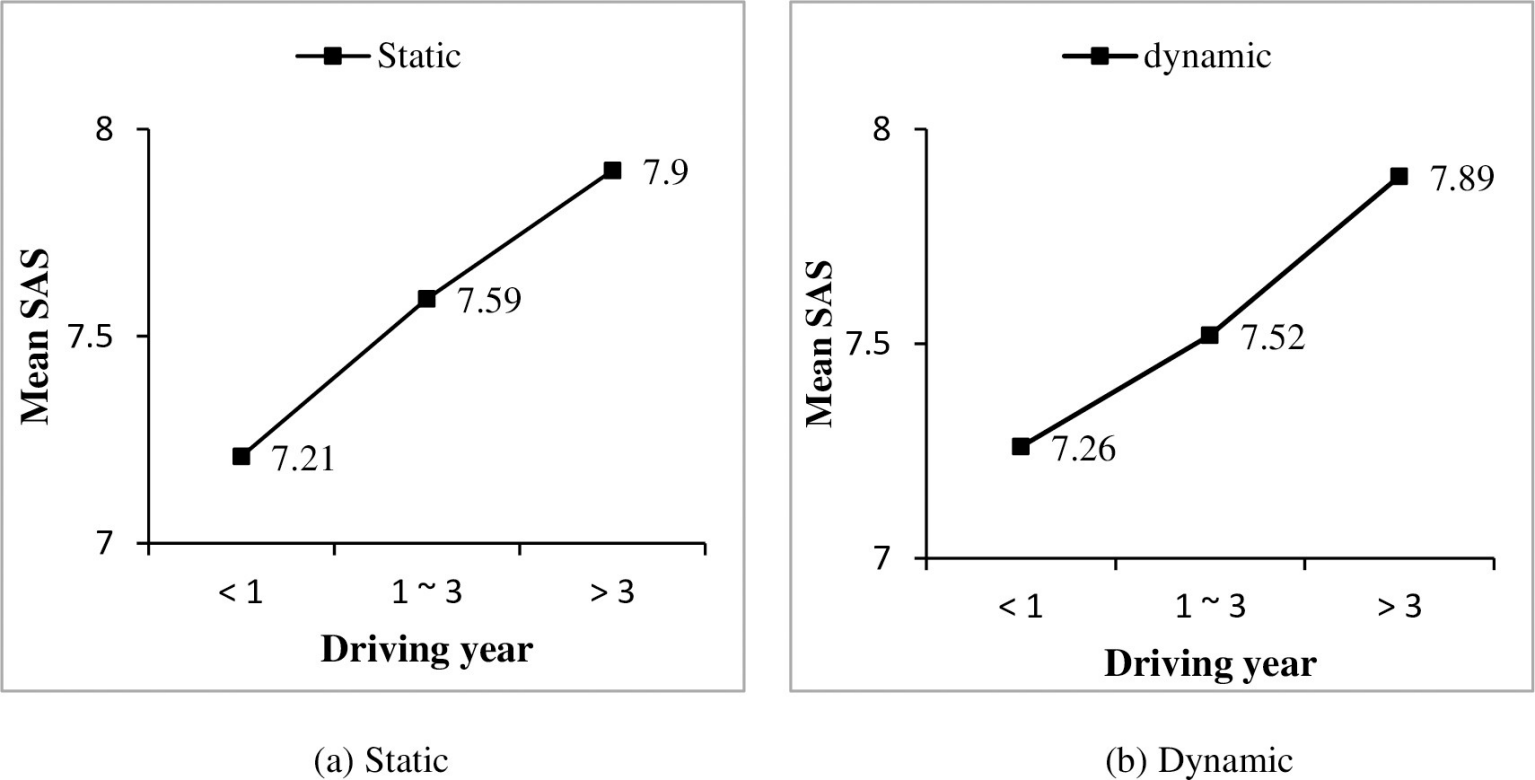
Effect of driving year on self-assessment scores (SAS) under different driving year conditions.

**Fig 3:**
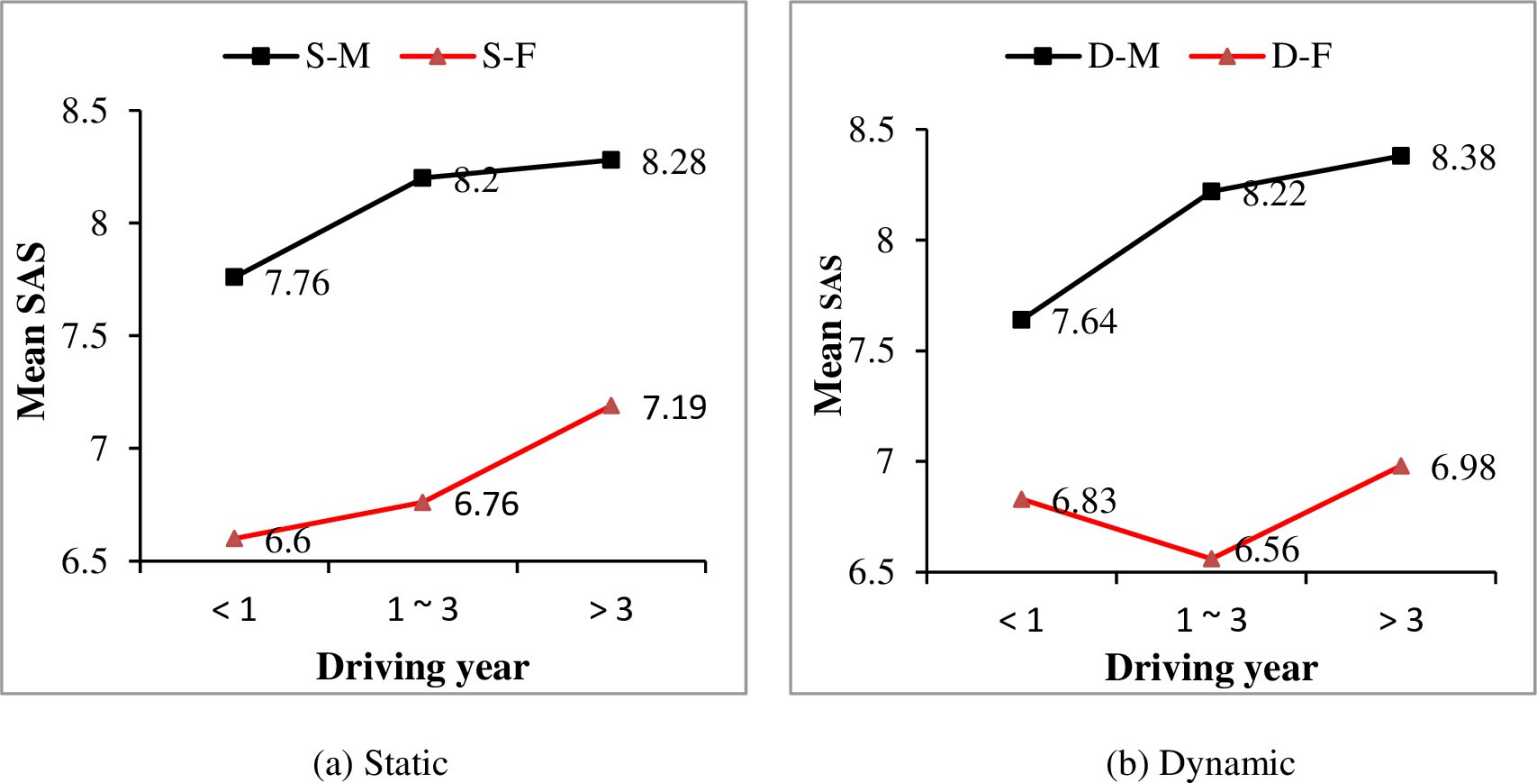
Effect of gender on self-assessment scores (SAS) under different driving year conditions.

**Fig 4:**
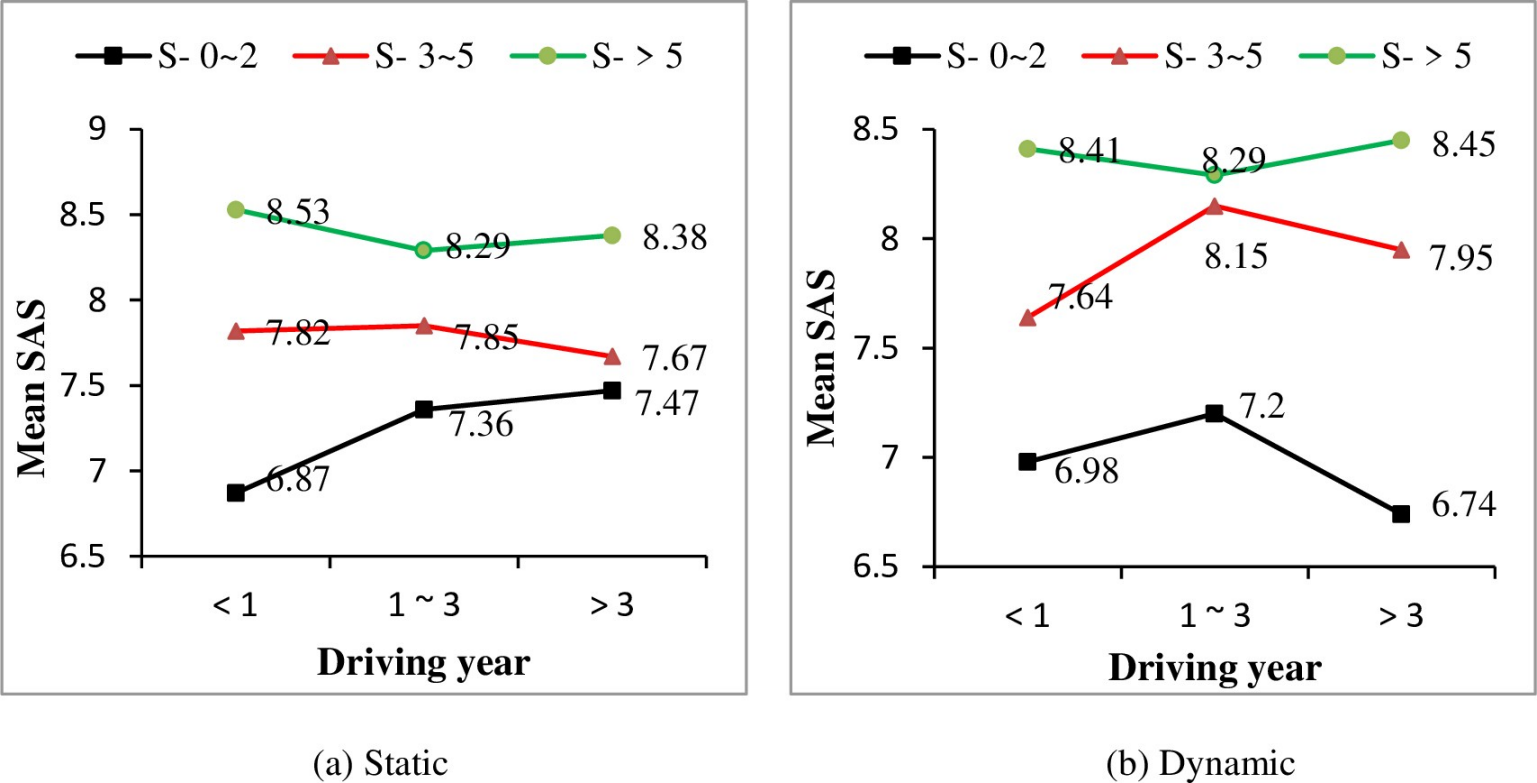
Effect of driving frequency on self-assessment score (SAS) under different driving year conditions.

**Fig 5:**
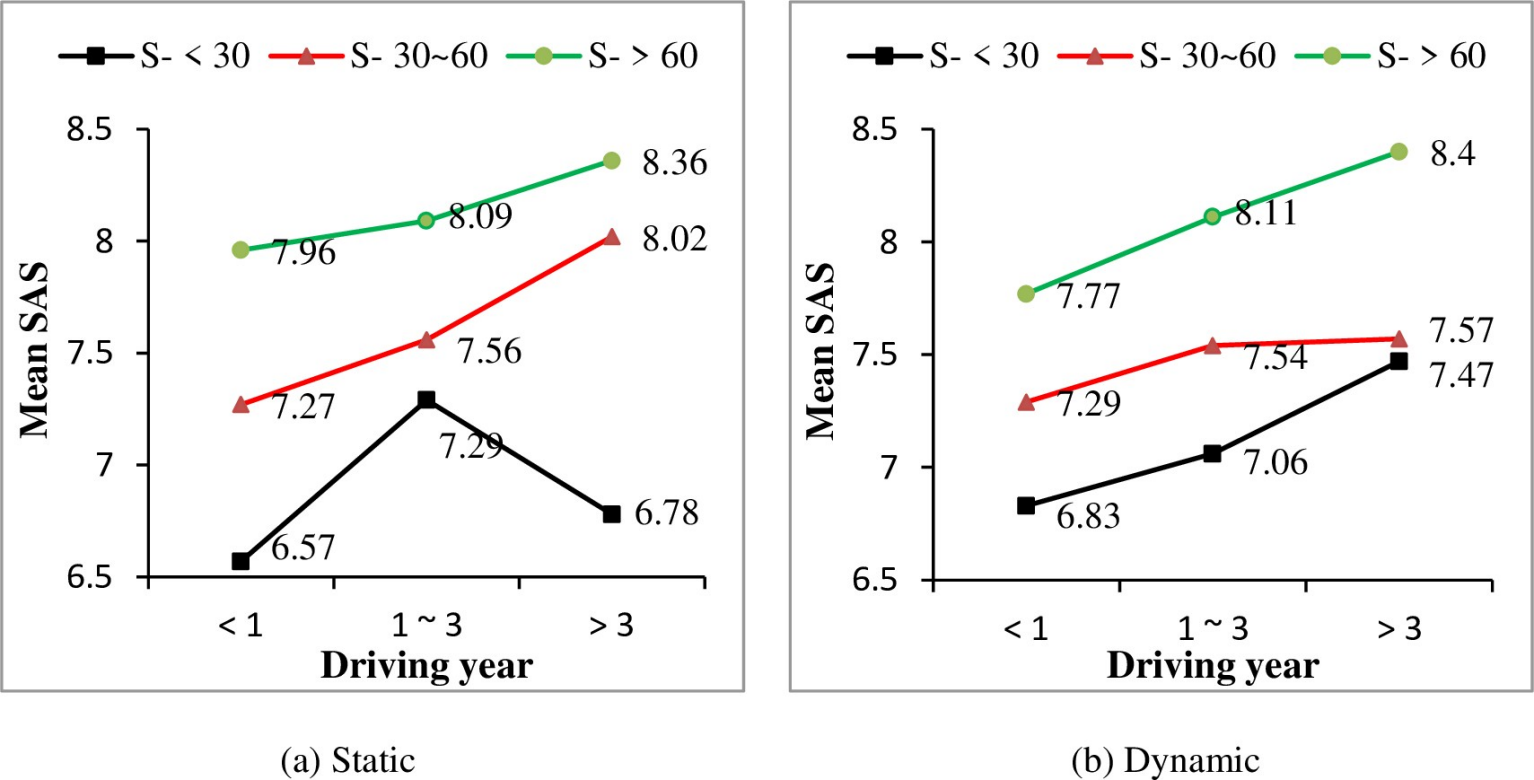
Effect of driving time on self-assessment scores (SAS) under different driving year conditions.

A one-way ANOVA was conducted between driving year and drivers’ observation ability self-assessment scores under both static information and dynamic information, respectively. Significant effects of driving year on SAS were found for both static information condition (F = 3.968, P<0.05) and dynamic information condition (F = 3.372, P<0.05). Generally, drivers’ SAS of observation ability increased with their driving years, as shown in Fig 2. Furthermore, effects of drivers’ gender, driving frequency and driving time on SAS were considered. For both static and dynamic conditions, male drivers’ SAS are higher than female drivers (see Fig 3). And with drivers’ driving year increased, both male and female drivers’ SAS increased, except for female drivers with driving experience of 1–3 years. Similarly, significant effects of driving frequency and driving time on drivers’ SAS were found for both static and dynamic conditions (show in Table 4). It was shown that with more practical driving experience, i.e., a higher driving frequency and more driving time, drivers’ SAS of observation ability increased as well (as shown in Figs 4 and 5, respectively).

**Table 4:** One-way ANOVA between drivers’ SAS and their characteristics.

Variables Classification			Gender	Driving year	Driving frequency	Driving time
**SAS**	Static	<1	9.931**	3.968*	5.780**	3.752*
		1~3	32.576**		3.631*	2.296
		>3	9.989**		3.279*	5.913**
	dynamic	<1	5.048*	3.372*	4.308*	1.778
		1~3	41.963**		5.901**	3.710*
		>3	16.753**		3.445*	6.559**

### 3.2 Mutual-assessment analysis

**Fig 6:**
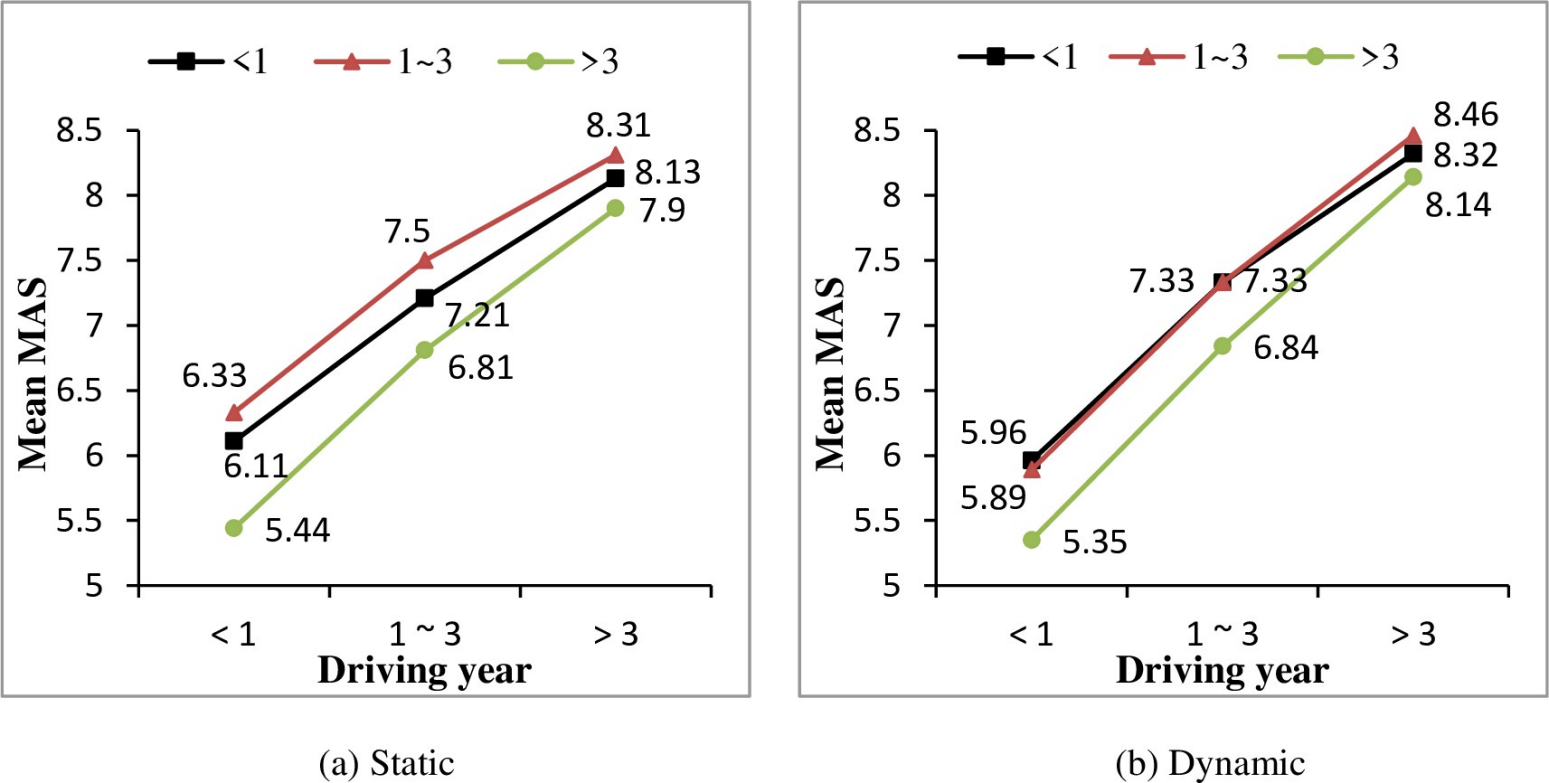
Effect of driving year on mutual-assessment scores (MAS) under different driving year conditions.

Analysis between driver characteristics and drivers’ MAS were conducted as well. As shown in Table 5, only driving year has a significant effect on MAS. Similar with the SAS results in Section 3.1, drivers’ MAS increased significantly with the growth of their driving years for both static and dynamic objects, as shown in Fig 6. However, for drivers in in “>3” group, no significant effect of driving year was found (F = 3.372, P>0.05).

**Table 5:** One-way ANOVA between MAS and driver characteristics.

Variables Classification			Gender	Driving year	Driving frequency	Driving time
**Mutual**	Static	**<1**	.338	7.128**	.651	.087
		1~3	.922	7.057**	.651	.092
		>3	1.406	2.319	.067	.195
	dynamic	**<1**	.264	3.442*	1.499	.932
		1~3	1.562	4.409*	.659	.564
		>3	3.897*	1.576	1.297	.273

### 3.3 Structural equation model

**Fig 7:**
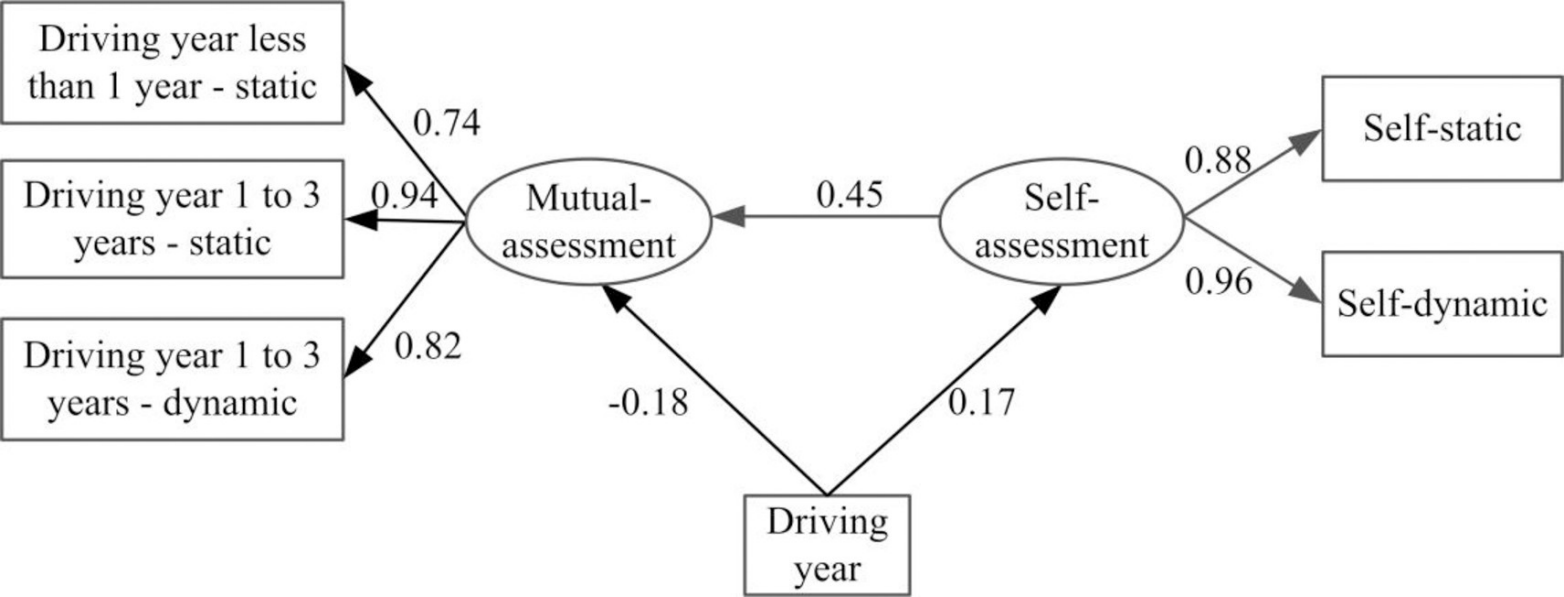
Structural equation model (statistically significant at p<0.05).

The basic structural correlation among all the variables is shown in Fig 7. The relationship between the hypothesized latent variables that affect the observation ability is quantified. Table 6 shows the definitions and input codes for the model. It was shown that driving year has a positive correlation effect on the self-rating score of drivers (the effect value is 0.36). The self-rating score of drivers with long driving year is higher than that of drivers with low driving year. However, driving year has a negative correlation effect on drivers’ MAS (the effect value is -0.38), and the scores given by drivers with long driving year is lower than that of drivers with low driving year. And drivers’ SAS has a positive correlation effect on MAS (the effect value is 0.45). The high self-assessment score of drivers also has a higher mutual assessment score. The SEM results are consistent with the results in section 3.1 and section 3.2.

**Table 6:** Definition of variables in SEM.

Latent factor	Observable variables
Driving years	Driving years (Within 1 year = 1,1 to 3 years = 2, Over 3 years = 3)
Self-assessment	Static observation ability
	Dynamic observation ability (signal and surrounding vehicles)
Mutual-assessment	Static observation ability (Within 1 year)
	Static observation ability (1 to 3 years)
	Dynamic observation ability (1 to 3 years)

To examine data fitting performance, a wide range of fit criteria were considered and listed in Table 7. It was shown that all the fitted variables met model requirements, which indicated that the model adopted in this study reach an acceptable fitness level.

**Table 7:** Fit statistics for SEM models.

Fit Index	Acceptable Fit	Model Value
CMIN/DF	<2	0.917
P-value	>0.05	0.451
RMR(Root mean square residual)	<0.05	0.009
GFI(Goodness of Fit Index)	>0.9	0.994
TLI(Tucker-Lewis index)	>0.9	1.000
IFI(incremental fit index)	>0.9	1.000
RMSEA(Root Mean Square Error of Approximation)	<0.1	0.000
CFI(Comparative Fit Index)	>0.9	1.000

### 3.4 Cognitive bias

#### 3.4.1 Cognitive bias of drivers under different driving year groups

**Fig 8:**
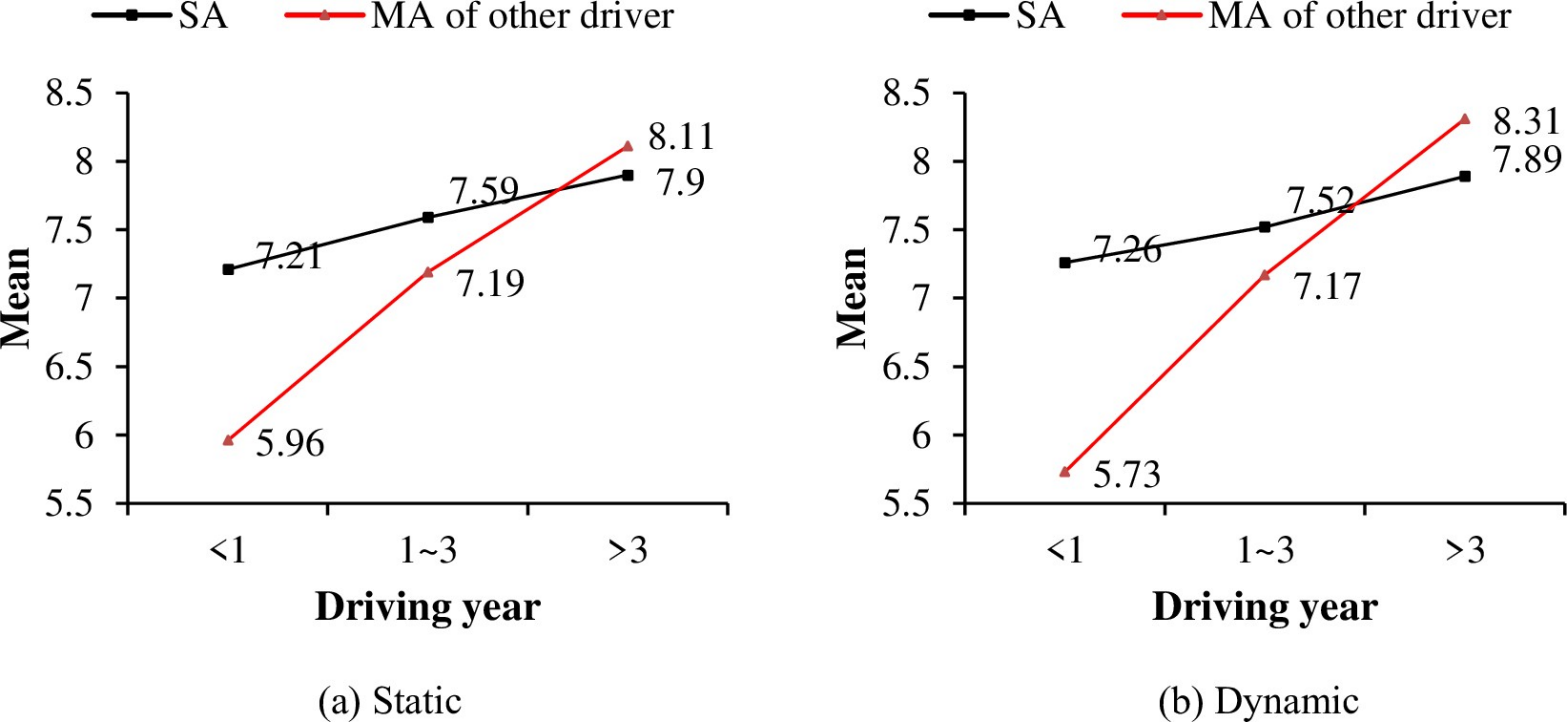
Comparisons of mean self-assessment and mutual-assessment score.

A T-test was adopted in this paper between drivers’ SAS and MAS to explore drivers’ cognitive bias. Significant difference was found for both static object (t = 11.97, P<0.01) and dynamic object (t = 13.34, P<0.01). Fig 8 shows the comparison under different driving year conditions. Drivers’ SAS is much higher than MAS when drivers are in “<1” and “1~3” driving year group. However, for the “>3” driving year group, drivers’ SAS is slightly lower than MAS.

**Fig 9:**
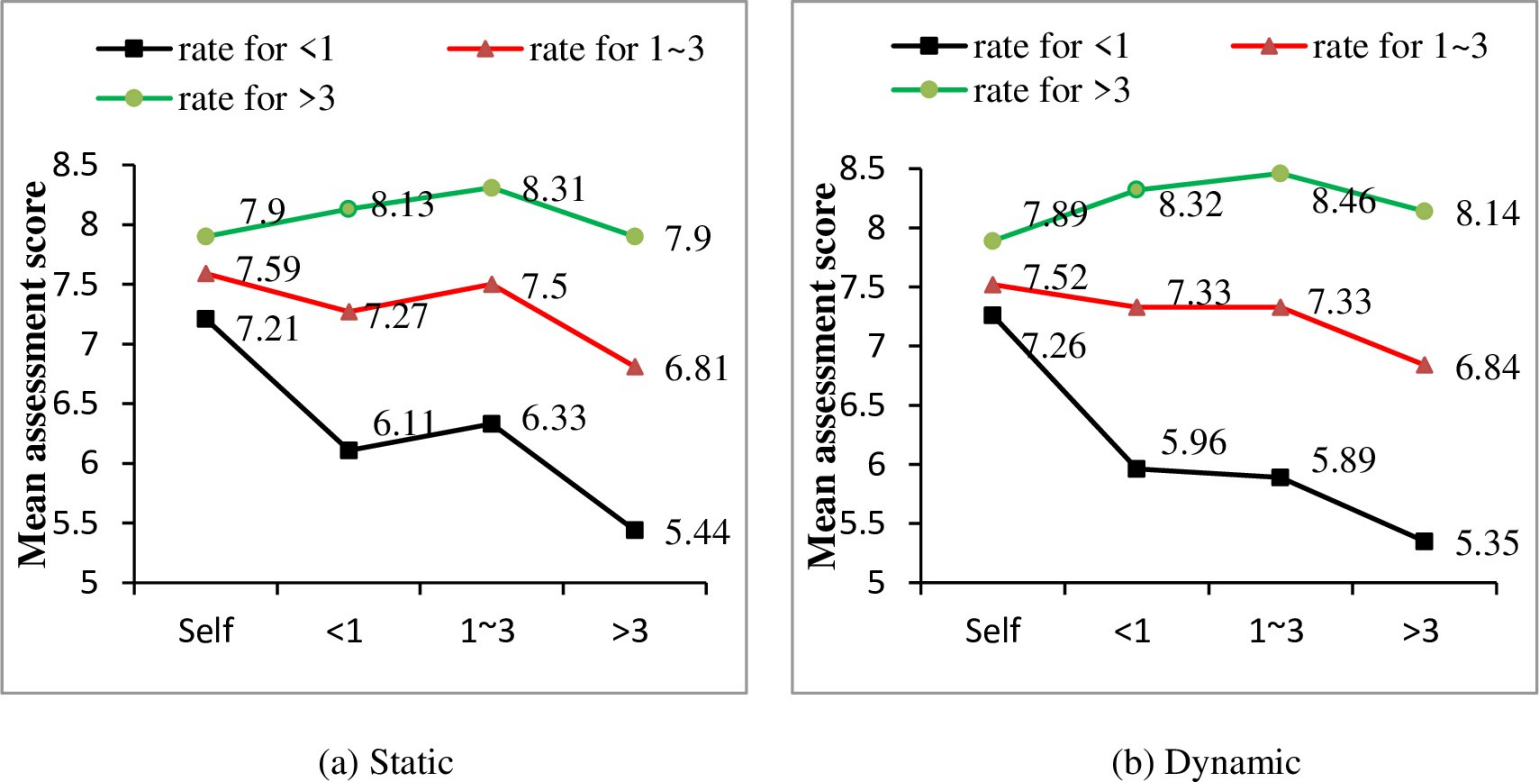
Comparison between self-assessment and mutual-assessment.

Detailed difference between drivers’ SAS and MAS in each driving year group was listed in Table 8 and Fig 9. For drivers with driving experience less than 1 year, their SAS are significant different from their MAS given by all driving year groups (e.g. “<1”→“<1”, “1~3”→“<1”, “>3”→“<1”). Drivers’ mean SAS of observation ability for static object in this group is 7.21, which is much higher than the MAS given by others, i.e., 6.11, 6.33 and 5.44, respectively. The same trend can be found for the dynamic objects’ observation ability. And the difference between SAS and MAS is greater when observe dynamic object than static object. For drivers those in driving year group “1~3”, significant difference from MAS was only found in driving year group “>3”, e.g., for static object, the T-test result was 12.662 (7.59 vs. 6.81), and for dynamic object, the T-test result was 9.741 (7.52 vs. 6.84). And for driving year group “>3”, no significant difference was found between drivers’ SAS and MAS given by the “>3” group. Different from driving year “<1” and “1–3” group, drivers’ mean SAS in this group is slightly lower than the MAS given by the “<1” and “1–3” group. For static object, drivers’ mean SAS is 7.9, while the MAS given by “<1” and “1–3” group are 8.13 and 8.31, respectively. Same results can be found for the dynamic object’s observation ability (7.89 vs. 8.32 and 8.46).

**Table 8:** Differences between drivers’ self-assessment and mutual-assessment.

Variables		Static	Dynamic
Self	Mutual		
<1	“<1” → “<1”	20.487**	28.532**
	“1~3” → “<1”	6.473*	9.067**
	“>3” → “<1”	17.760**	26.495**
1~3	“<1” → “1~3”	3.301	1.109
	“1~3” → “1~3”	.255	1.178
	“>3” → “1~3”	12.662**	9.741**
>3	“<1” → “>3”	1.235	4.508*
	“1~3” → “>3”	4.780*	9.536**
	“>3” → “>3”	.000	1.092

** At 0.01 significance level.

* At 0.05 significance level.

The mutual-assessment difference was investigated in this study as well. Both assessors and assessed objects were divided by three driving years group (e.g. “<1”, “1~3”, “>3”). Mutual-assessment scores could be analyzed from two aspects (See Table 9 and Fig 9). For same assessors, drivers MAS given to others in different driving year groups are significantly different. Drivers’ MAS increased significantly with the growth of their driving years for both static and dynamic objects, as shown in Fig 9. For same assessed objects, drivers’ MAS given by others are significantly different when the assessed objects are drivers in “<1” group and “1~3” group. Drivers in “>3” group gave the lowest scores, drivers in “<1” group gave the intermediate scores, while drivers in “1~3” group gave the highest scores. Drivers’ MAS given by others are not significantly different when the assessed objects are drivers in “>3” group.

**Table 9:** Mutual-assessment difference among different driving year groups.

Same assessor			Same assessed objects		
Groups	Static	dynamic	Groups	Static	dynamic
“<1” → “<1” “<1” → “1~3” “<1”→ “>3”	63.474**	88.258**	“<1” → “<1” “1~3” → “<1” “>3” → “<1”	7.128**	3.442*
“1~3” → “<1” “1~3” → “1~3” “1~3” → “>3”	72.047**	125.184**	“<1” → “1~3” “1~3” → “1~3” “>3” → “1~3”	7.057**	4.409*
“>3” → “<1” “>3” → “1~3” “>3” → “>3”	43.892**	56.815**	“<1” → “>3” “1~3” → “>3” “>3” → “>3”	2.319	1.576

** At 0.01 significance level.

* At 0.05 significance level.

#### 3.4.2 Meta-analyses of cognitive biases

In this section, results obtained in this paper were compared with previous studies, from the aspects of novice drivers’ observation ability. As shown in Table 10, drivers’ driving ability and behavior are known to be affected by individual factors, such as gender, driving year, driving frequency, driving times etc. [15, 16, 22]. In this paper, gender, driving year, driving time and driving frequency all have significant effects on drivers’ observation abilities, for both static and dynamic objects. Consistent with previous studies, male drivers’ observation ability scores were higher than female drivers. Generally, drivers with more practical driving experience (e.g., longer driving year or more driving times) has better observation abilities than drivers with less driving experience.

**Table 10:** Meta-analyses of cognitive biases of influencing factors.

Variable	Reference		This paper
	Result	Author	Result
Gender	Male’s observation ability score higher than female	[23]	Consistent
Driving Year	Driver with longer driving year has better observation ability	[24–26]	Consistent
Driving Frequency	Driver with higher driving frequency has better static observation ability	[27]	Consistent
Driving Times	Driver with longer driving times has better observation ability	[25]	Consistent

Table 11 shows the cognitive bias between self-assessment and mutual assessment. The degree of cognitive bias between self-assessment and mutual assessment depends on the comparison method used to evaluate novice drivers (mean, ordinary observer, expert). In this paper, the proportion of novice drivers who overestimate their observation ability is lower than that of previous studies, which may be due to inconsistent comparison methods. The mutual assessment data are novice drivers’ assessment of other novice drivers, which is an average comparison.

**Table 11:** Meta-analyses of cognitive bias between self-assessment and mutual assessment.

Reference		This paper
Author	Result	Result
[28]	95% self-assessment score was higher than the mutual assessment score of experts 5% self-assessment was lower than the mutual assessment of experts	76.45% self—assessment is confident 33.55% self—assessment is not confident. Indicating novice drivers are more likely to overestimate themselves
[29]	75% self-assessment score is confident 25% self-assessment is not confident	
[30]	Novice drivers are more likely to overestimate themselves	
[31]	significant positive correlation between self-assessment and examiner assessment	
[32]	Self-assessment and mutual assessment are negatively correlated	
[33]	no significant relationship between self-assessment and objective assessment	
[34]	The correlation is poor between self-assessment and actual performance	

## 4. Discussion

For drivers’ self-assessment, gender, driving year, driving frequency and driving time all had significant effects on drivers’ SAS of observation abilities. Generally, male drivers are more confident than female drivers with higher SAS. And drivers with more practical driving experience (longer driving year, higher driving frequency and more driving time) are more confident than drivers driving less. All the findings obtained in this study were consistent with previous studies [23, 25–27].

As for drivers’ mutual-assessment, no significant effects of drivers’ gender, driving frequency and driving time on MAS were found. But drivers’ MAS for both static and dynamic objects increased with the rising of driving year, which has been verified by previous studies [24, 26]. For both self-assessment and mutual-assessment, significant difference can be found for different driving year conditions. Thus, a SEM model was adopted in section 3.3. And the results of the SEM model also proved it.

Cognitive biases are tendencies commonly used to acquire and process information by filtering it through one’s own beliefs and experiences [35]. The difference between SAS and MAS, and also difference between MAS and MAS, both could be regarded as cognitive bias. According to the results in section 3.4, two kinds of cognitive bias can be obtained.

The first cognitive bias is the overconfidence induced by novice drivers. For the driving year “<1” group, their SAS were much higher than the MAS. Studies have shown that drivers with low driving ability are less accurate in self-assessment than those with high driving ability [36]. If the mutual-assessment scores of experienced drivers (i.e., driving year “>3” group) are objective, 76.45% of novice drivers are overconfident, especially for the driving year “< 1”group In other words, drivers with driving experience less than one year in “<1” group consider that although their observation ability is lower than drivers with more driving years, but themself is the outstanding person among drivers with equivalent driving experience.

The second cognitive bias is the irregular mutual-assessment of drivers in “1~3” group. Generally, drivers’ mutual-assessment scores given ought to show a certain trend, for example liking the sustained growth trend of self-assessment scores following the increase of driving year. However according to the comparison of drivers’ mutual-assessment scores given, drivers in “1~3” group gave the most optimistic assessment. And drivers in “>3” group gave the most conservative assessment (even less than drivers in “<1” group), which is consistent with their conservative driving behavior and safer vehicle maneuvers of experience drivers [37].

One cause of cognitive bias is that respondents wanted to present their positive views to the researchers even though considering themselves no higher than average [38]). The form of online survey in this paper could weaken this effect because lacking of the face-to-face mental stress. More likely, the inaccurate self-assessment of observation ability was caused by the lack of accurate cognition of driving ability [36]. This can well explain the first cognitive bias of novice drivers’ overconfident in this paper.

For the irregular mutual-assessment of drivers in “1~3” group, it showed their most optimistic attitude for driving. However, although drivers in “1~3” group involved in fewer traffic accidents (17.2% vs. 18%), involved more casualty accidents (16.7% vs. 15%) than drivers in “<1” group [39]. This may be caused by their incomplete driving experience. When novice drivers started driving, they used to keep extremely conservative driving behavior because of the fear of unknown driving environment, such as low speed and narrow visual field [40, 41]. This makes novice drivers always faced relatively simple driving tasks. High frequent visual stimuli of relative low risk driving tasks could make them skillfully observe general driving scenarios, which may let them believe an illusion of completely controllable. In other words, general confident rising may lead to drivers in “1~3” group feel that driving is an easy job. In fact, there are some complex driving scenarios they did not meet, such as the sudden intrusion of surrounding vehicle into their trajectory.

**Fig 10:**
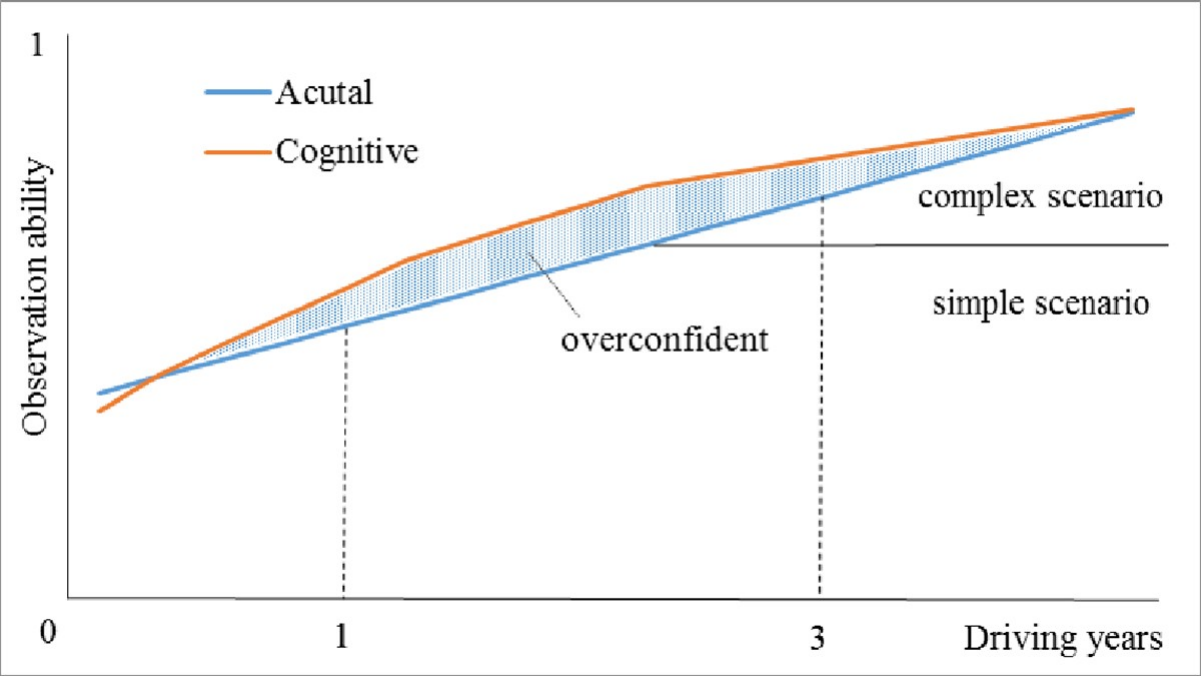
Observation ability change schematic following the rising of driving years.

Based on above analysis, we tried to describe the changing mechanism of novice drivers’ observation ability following the rising of driving years (See Fig 10). Novice drivers are general overconfident, especially for drivers in “1~3” group. Regular driving practice will rapidly improve novice drivers’ observation ability for simple scenario. As the rising of confident, novice drivers gradually try a more difficult driving task, such as higher speed and more lanes change [42]. Then they may meet part complex scenario which corresponds to a higher need for observation. The observation difficult improvement will slow the growth trend of self-confidence, until it gets closer and closer to actual observation. Even part experienced drivers may less self-confident after coping with more and more complex scenarios, especially seeing or involving in a traffic accident.

What can we do to improve novice drivers’ driving safety? We can provide solutions from three aspects. One is to reduce their cognitive observation ability. Provide driver feedback is a feasible method can help them overcome the pilot inaccurate self-assessment, now the European countries have the self-assessment feedback in driver training, as part of the driver’s license system [43]. For example, after some unsuccessful driving process, driving scene reproduction to novice driver could make them know their weaknesses clearly. The other is to improve their actual observation ability. Training is the feasible method, especially for complex scenarios novice drivers not often to meet [44]. For example, few novice drivers could deal with emergency collision avoidance successfully because the frequency of incident is very low in the real world, but repeating training can improve emergency response performance. Finally for traffic management department, extensive publicity and education to novice drivers is essential and effective. In addition, new traffic management technology (e.g. active luminous signs) and mobile on-board equipment (e.g. intelligent warning) are also worthy of consideration to assist novice drivers.

## 5. Conclusion

The first three years after novice drivers getting driving license is the critical improving period to gain driving experience and ability, and is the unsafe period easily involved in an accident. It is crucial whether novice drivers have accurate cognition for their driving abilities, especially observation abilities of static and dynamic traffic information while driving. This paper analyzed drivers’ self-assessment and mutual-assessment scores among different driving years based on online survey data, and results shown that two cognitive biases for novice drivers’ observation abilities occurred. The first cognitive bias was the general overconfidence induced by novice drivers. The second cognitive bias was the highlighted risk for drivers in “1~3” group because of their incomplete driving experience. And then observation ability change schematic following the rising of driving years was tried to present to guide the safety improving solutions for novice drivers. However, subjective survey results could describe the change trend, but not enough to dissect the specific possible causes and influence degree. Therefore, the next study will use driving simulator and eye-track to test novice drivers’ observation ability under detail driving scenarios, and then find their definite deficiency based on hierarchical driving observation difficulty (simple, complex, emergency).

## Supporting information

S1 Appendix Questionnaire. (DOCX)

S1 Dataset All data underlying the findings. (ZIP)
