# Supplementary material for: Cognitive bias analysis of young novice drivers’ observation abilities—A questionnaire-based study
Source: PLoS One. 2021 May 11;16(5):e0251195. doi: 10.1371/journal.pone.0251195 (PMC8112646; doi:10.1371/journal.pone.0251195)
Supplement: S1 Appendix — (DOCX) [file pone.0251195.s001.docx]

**Investigation on observation ability of novice drivers**

Dear Sir /Madam：

Hello! We are conducting a survey on novice drivers to accurately analyze the current driving observation ability for the improvement of novice drivers' driving ability. Your comments are very valuable for our research. We sincerely appreciate your cooperation. Please answer the following questions in order according to your actual feelings. Thank you very much!

1. Your gender is？

A.Man B.Woman

2. Do you have a driver's license ？

A.Yes B.No

3. The type of your driver's license is？

A.C1（Compact car） B.C2（Automatic car） C.C3（Low-speed truck） D.Else

4. Do you have a car？

A.Yes B.No

5. How often do you drive after your driving license obtained？

A.Less than once a week B.One to Two times per week

C.Three to Five times per week D.More than Five times per week

6. How long time of driving for your single travel？

A. Less than 30 minutes B. 30-60 minutes

C. 60-120 minutes D. Longer than 120 minutes

7. Have you ever been involved in a traffic accident？

A. Zero B. One to Two times

C. Three to Five times D. More than Five times

8. If you can observe traffic sign (such as directional sign and stop sign) timely when driving ? Please fill in score (0-10)

9. If you can observe traffic marking (such as double amber lines and lane divider) timely when driving ? Please fill in score (0-10)

10. If you can observe traffic signal light switching at intersection timely when driving ? Please fill in score (0-10)

11. If you can observe surrounding vehicle (such as location and space) accurately when driving ? Please fill in score (0-10)

12. Please rate the observation ability of **other drivers** with different driving experience (score 0-10) :

| Driving experience | Static observation | | Dynamic observation | |
| --- | --- | --- | --- | --- |
|  | traffic sign | traffic marking | traffic signal light | surrounding vehicle |
| <1 driving year |  |  |  |  |
| 1~3 driving year |  |  |  |  |
| >3 driving year |  |  |  |  |
